# Hot Deformation Behavior and Microstructural Evolution of a Novel β-Solidifying Ti–43Al–3Mn–2Nb–0.1Y Alloy

**DOI:** 10.3390/ma12132172

**Published:** 2019-07-06

**Authors:** Qianqian Wu, Ning Cui, Xiaohong Xiao, Xiaopeng Wang, Ertuan Zhao

**Affiliations:** 1 School of Mechanical and Automotive Engineering, Qingdao University of Technology, Qingdao 266520, China; 2 State Key Laboratory of Advanced Welding and Joining, Harbin Institute of Technology, Harbin 150001, China; 3 School of Mechanical Engineering, Shandong University of Technology, Zibo 255000, China

**Keywords:** TiAl alloys, β phase, hot deformation, processing map

## Abstract

In this paper, the hot deformability and mechanical properties of a novel Mn- and Nb- containing TiAl alloy were studied systematically with the use of isothermal compression experiments. The results show that the alloy has low deformation resistance and a low activation energy (392 KJ/mol), suggesting that the alloy has good hot deformability. A processing map was established, which shows that the present alloy has a smaller instability region and wider hot working window compared with other TiAl alloys. Microstructural observation shows that the initial lamellae completely transformed into fine equiaxial γ grains when the alloy was compressed at 1200 °C/0.01 s^−1^, which corresponds to the optimum deformation condition. Based on the above results, an intact TiAl billet was successfully fabricated by one-step large deformation using a four-column hydraulic machine. The microstructure of the billet is almost completely composed of recrystallized γ grains with large angle boundaries. Tensile testing shows the billet exhibits high tensile strength (780 MPa) and high elongation (1.44%) simultaneously, which benefits from fine γ grains with an average size of 4.9 μm. The ductile–brittle transition temperature is between 750–800 °C.

## 1. Introduction

TiAl alloys are promising substitutes for Ni-based superalloys in aero-engines because of their advantages of being lightweight and having superior high temperature strength [1,2,3]. Although the research on TiAl alloys has made considerable progress in recent decades, some questions still remain to be solved, such as intrinsic brittleness and low hot deformability [4]. Thermomechanical treatment (TMT) can effectively refine the microstructure and enhance the ductility. Previous studies have shown that a disordered β phase can be introduced into the high-temperature microstructure by adding β stabilizers, which can significantly enhance the hot deformability of TiAl alloys. Thus, novel β–solidifying TiAl alloys have become a hot topic of research.

The properties of β–solidifying TiAl alloys are highly sensitive to their alloy composition, especially β stabilizers. Extensive study has been conducted on several typical β–solidifying TiAl alloys. The TNM (Ti–43.5Al–4Nb–1Mo–0.1B) alloy exhibits relatively balanced mechanical strength and ductility [5]. High–Nb containing TiAl alloys have good creep resistance and high-temperature strength [6,7]. The excellent high-temperature performance is ascribed to the solid-solution hardening caused by Nb additions [8,9]. The Ti–43Al–9V–Y alloy has superior hot deformability and high room temperature elongation [10]. V has strong β stability. A large amount of the β phase can precipitate in the Ti–43Al–9V–Y alloy, which can enhance the deformability of alloys. High elongation is related to the low hardness of the V-rich β phase [11]. However, there are not many applications for these TiAl alloys yet because they have their own disadvantages. Only the TNM alloy has been applied to the PW1134G engine for the Airbus A320 [12]. A high V content is detrimental to the oxidation resistance. A uniform dense Al_2_O_3_ layer is key to improving the oxidation resistance of TiAl alloys. However, the Al_2_O_3_ layer formed in the high-V-containing TiAl alloy is thin and discontinuous [13,14]. A high Nb content limits the hot deformability of alloys due to the weak β stability of Nb. Thus, it is necessary to further develop novel β–solidifying TiAl alloys with good hot deformability and balanced mechanical properties. Previous studies have shown that Mn is a typical ductile element. Mn tends to occupy the Al position in the crystal lattice, and reduce the lattice tetragonality of the γ phase. This can promote the dislocation slip and enhance the ductility of alloys [15]. Mn is also a strong β stabilizer, which is beneficial for the hot deformability of alloys [16]. The mechanical strength and high temperature properties of alloys can be improved by adding Nb due to solid-solution hardening [6]. Moreover, a large number of studies have shown that Y has a strong affinity with oxygen and can be used to remove oxygen in TiAl alloys [17]. The Y-rich phase (Y_2_O_3_) tends to precipitate at phase boundaries, and thereby prevents grain growth [18,19]. Both lamellar colony size and lamellar spacing can be refined by Y [18]. It is predicted that more balanced properties may be obtained by adding a moderate amount of Nb, Mn, and Y simultaneously. To date, very little research has been conducted on the Mn- and Nb-containing TiAl alloy.

In this study, the hot deformability of a novel Mn- and Nb-containing TiAl alloy was evaluated systematically by isothermal compression tests. The microstructural evolution of the alloy during thermal compression was investigated with the aid of a processing map. Furthermore, a crack-free as-forged billet was successfully prepared by a one-step large deformation. The tensile properties of the as-forged billet were also studied.

## 2. Experimental Procedures

An ingot of Ti–43Al–3Mn–2Nb–0.1Y alloy (Φ 110 mm × 150 mm) was fabricated by vacuum induction melting. Chemical composition analysis showed that the actual composition was Ti–43.2Al–2.94Mn–2.1Nb–0.13Y. Several cylindrical specimens (Φ 8 mm × 12 mm) for hot compression were cut from the alloy and then polished smooth with fine sandpaper. Isothermal compression experiments were carried out by a Gleeble 1500D simulator (DSI, Saint Paul, MN, USA). According to experimental results, a Ti–43Al–3Mn–2Nb–0.1Y billet (Φ 140 mm × 13 mm) was produced by one-step near-isothermal canned forging using a four-column hydraulic machine, followed by furnace cooling. Tensile properties were measured using an Instron tensile testing machine. Microstructures of the as-cast ingot and compressed specimens were examined by scanning electron microscopy (SEM, FEI, Hillsboro, OR, USA) and transmission electron microscopy (TEM, FEI, Hillsboro, OR, USA). The microstructure of the as-forged billet was analyzed by electron back-scattered diffraction (EBSD, FEI, Hillsboro, OR, USA). Samples for TEM and EBSD were prepared using a standard procedure, which has been described in previous research [20].

## 3. Results and Discussion

### 3.1. Initial Microstructure

**Figure 1:**
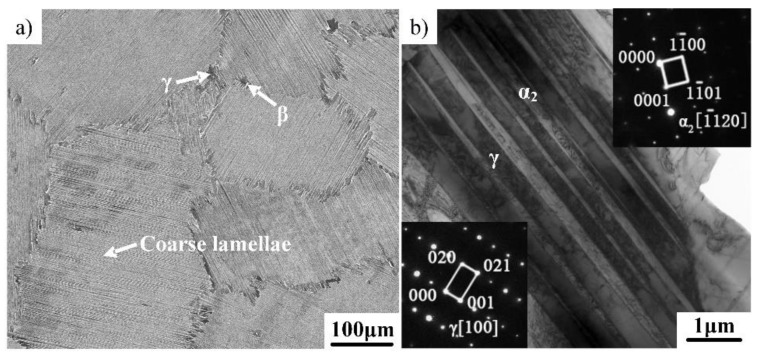
Microstructural characterization of the Ti–43Al–3Mn–2Nb–0.1Y ingot. (**a**) SEM images and (**b**) TEM images.

Figure 1a exhibits an SEM image showing the initial microstructure of the Ti–43Al–3Mn–2Nb–0.1Y ingot, which mainly consists of coarse γ/α_2_ lamellar colonies with an average size of above 200μm. The γ/α_2_ lamellae were further confirmed by selected area electron diffraction (SAED) patterns, as shown in Figure 1b. Coarse lamellae are detrimental to the tensile ductility of the Ti–43Al–3Mn–2Nb–0.1Y alloy. Some white phase and globular black phase can also be found at the boundaries of γ/α_2_ lamellae. Energy dispersive X-ray spectrometry (EDX) shows that the contents of Ti, Al, Mn, Nb, and Y in the black phase are 48.13 at%, 48.38 at%, 2.23 at%, 1.15 at%, and 0.11 at%, respectively, which confirmed that the black phase is γ-TiAl. The contents of Ti, Al, Mn, Nb, and Y in the white phase are 49.15 at%, 39.57 at%, 8.05 at%, 3.19 at%, and 0.04 at%, respectively. A high β-stabilizer content confirmed that the white phase is β. The high-temperature disordered β phase can enhance the hot deformability of TiAl alloys. However, the β phase transforms into the ordered β_0_ phase when the temperature drops below 1100 °C. In this study, no distinction in notation is made, and both are represented by β. Moreover, the precipitation of the globular γ phase around the β phase is owed to the phase transition α→β+γ during the solidification process [21].

### 3.2. Flow Behavior and Hot Deformability

**Figure 2:**
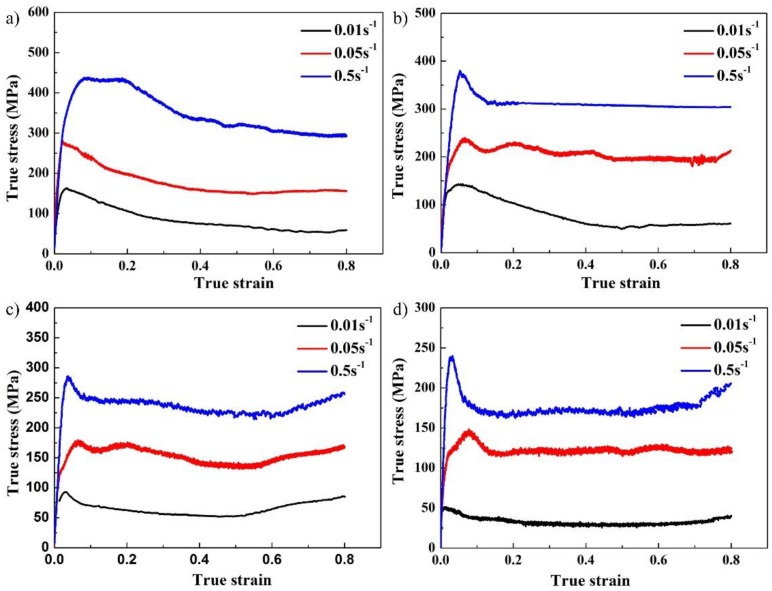
Isothermal compression curves of the Ti–43Al–3Mn–2Nb–0.1Y alloy. (**a**) 1100 °C, (**b**) 1150 °C, (**c**) 1200 °C, and (**d**) 1250 °C.

Isothermal compression experiments were conducted at different temperature and strain rates to study the hot deformability of the Ti–43Al–3Mn–2Nb–0.1Y alloy. Compression curves obtained at 1100–1250 °C/0.01–0.5 s^−1^ are exhibited in Figure 2. All curves exhibit a typical flow softening feature [22]. The temperature and the strain rate have a great influence on the peak stress. When the temperature and strain rate were specified, the stress had an evident upward trend in the early stages of the deformation, which is caused by the formation of a high density of dislocations. With increasing strain, some softening mechanisms may occur at high temperatures, such as adiabatic heating, dynamic recovery (DRV) and dynamic recrystallization (DRX) [23], leading to a decrease in the compressive stress. For example, adiabatic heating processes are those in which deformation-generated heat does not have sufficient time to dissipate at higher strain rates during hot deformation. This can lead to a temperature increase in the deformed specimens, which contributes to flow softening. Moreover, dynamic recovery can be promoted by adiabatic heating [24]. Semiatin et al. [25,26] have also confirmed that adiabatic heating contributes to flow softening by experiments. Furthermore, under the same conditions, the deformation resistance of the Ti–43Al–3Mn–2Nb–0.1Y alloy is lower than that of high-Nb containing TiAl alloys. For example, when Ti–43Al–3Mn–2Nb–0.1Y alloy deformed at 1200 °C/0.01 s^−1^, the corresponding peak stress is about 93 MPa, while the peak stress is about 150 MPa for the Ti-44Al-8Nb-0.2W-0.2B-0.1Y alloy. This indicates that the Ti–43Al–3Mn–2Nb–0.1Y alloy exhibits better hot workability [27].

Based on the above flow stress data, a thermodynamic calculation was also conducted. It is well known that the Arrhenius formula can be used to describe the dependence of flow stress on strain rate and temperature [28]. Mathematical derivation was carried out based on the Arrhenius formula. The Zener–Hollomon parameter (Z) can be expressed as Equation (1), the physical meaning of which is the temperature-compensated strain rate parameter. Z can be used to describe the combined effect of strain rate and temperature on the deformation process [29,30].

(1) $$Z=\dot{\varepsilon }\exp\left(Q/RT\right)=A{\left\{\sinh\left(\alpha \sigma \right)\right\}}^{n}$$

where $\dot{\varepsilon }$ is the strain rate, *Q* is the deformation activation energy, *R* is the gas constant, *T* is the temperature, *A* and *α* are material constants, *σ* is the flow stress, and *n* is the stress exponent.

The deformation activation energy defines an energy barrier that needs to be overcome by the thermal motion of atoms during hot deformation. It reflects the hot workability of alloys. The *Q* value can be obtained by the following equation:

(2) $$Q=R{\frac{\partial \ln\left[\sinh\left(\alpha \sigma \right)\right]}{\partial \left(1/T\right)}|}_{\dot{\varepsilon }}{\frac{\partial \ln\dot{\varepsilon }}{\partial \ln\left[\sinh\left(\alpha \sigma \right)\right]}|}_{T}$$

Based on the above formulas, the *Q* value of the Ti–43Al–3Mn–2Nb–0.1Y alloy is determined as being 392 kJ/mol [31]. The value is higher than that of Ti self-diffusion and Al self-diffusion in γ–TiAl. This indicates that the microstructural evolution was not controlled by the self-diffusion of Al and Ti atoms. Solid-solution strengthening and lattice distortion can be caused by adding Mn and Nb, which increase the deformation activation energy. Moreover, the *Q* value of the present alloy is lower than that of the high-Nb-containing alloy (494 kJ/mol) [27] and TNM alloy (524.67 kJ/mol) [32], indicating that the thermally activated process occurs more easily for the present alloy. This explained why the present alloy exhibits low deformation resistance.

### 3.3. Processing Map

**Figure 3:**
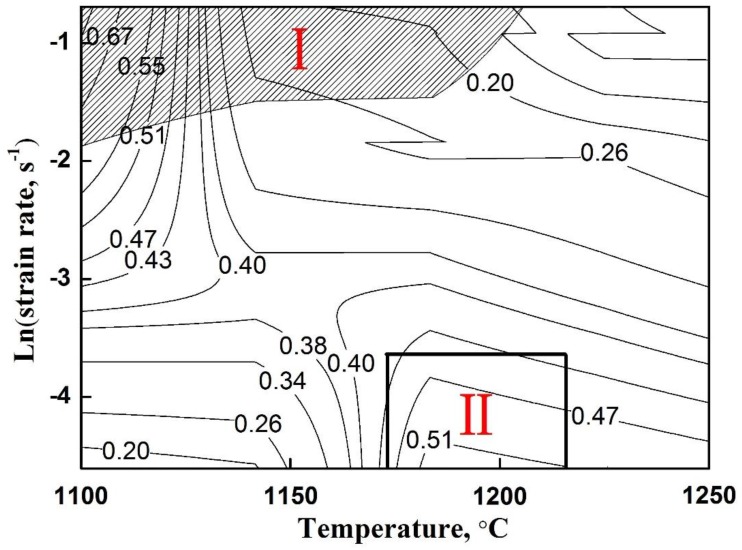
Processing map of the Ti–43Al–3Mn–2Nb–0.1Y alloy.

The plastic working of TiAl alloys is very difficult due to their disadvantage of intrinsic brittleness. It is necessary to obtain accurate hot working windows. A hot processing map is a reliable method to evaluate the hot deformability, analyze the deformation mechanism, and determine the deformation conditions. From the map, we can find various domains, which correspond to different deformation mechanisms [27,33]. The basic principle and construction method of the processing map have been introduced in earlier papers [34]. A processing map (Figure 3) of the Ti–43Al–3Mn–2Nb–0.1Y alloy was constructed based on the flow stress data obtained at a strain of 0.7. The number in the map represents the efficiency of power dissipation, which reflects the ratio of microstructural dissipation and total energy. Evidently, a high efficiency value means that more energy can be used for the microstructural evolution, which is desirable for the hot deformation. As can be seen from the processing map, two typical regions can be identified. The shadow region Ⅰ, occurring at a high strain rate (0.5 s^−1^) and low temperature (1100–1200 °C), represents the instability region, in which cracking, flow localization, and an adiabatic shear band may occur. Thus, the shadow region is undesirable for hot deformation of the alloy. Compared with high-Nb-containing TiAl alloys, the present alloy exhibits a smaller instability region [27], which is consistent with the results of the flow stress. It can be found that the instability region also exhibits high peak efficiency, which is due to cracking, etc., which would expend a lot of energy. Moreover, the map exhibits a high peak efficiency domain Ⅱ (called stability domain), which appears at 1200 °C/0.01 s^−1^. The stability domain generally represents the optimum deformation condition, which corresponds to certain softening mechanisms, including DRV, DRX, and superplasticity. Previous studies show that the deformation of TiAl alloys are generally conducted at 1250–1300 °C to avoid cracking [35]. This indicates that the hot working window of the Ti–43Al–3Mn–2Nb–0.1Y alloy was broadened due to the addition of Mn and Nb. It should be noted that the processing parameter obtained by the processing map is a rough range. The optimum hot processing condition and deformation mechanisms still need to be further verified by combining with the subsequent microstructural analysis.

**Figure 4:**
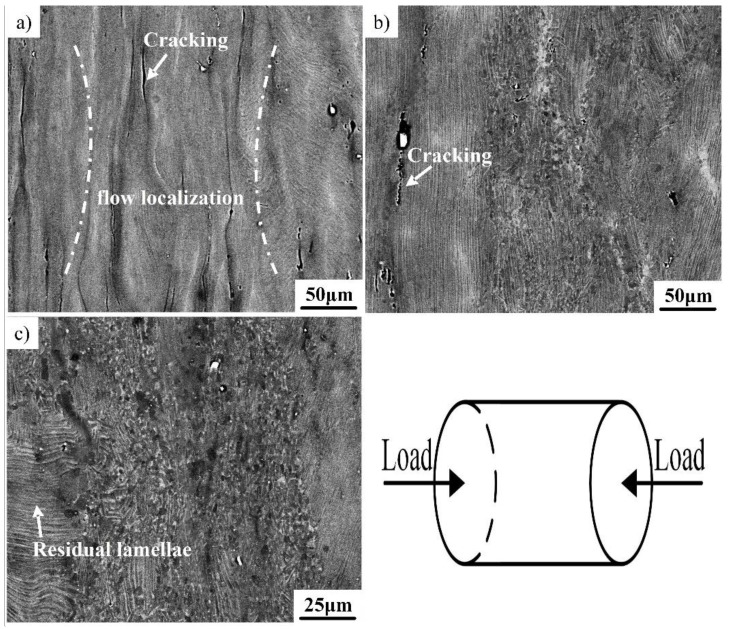
The microstructure of the Ti–43Al–3Mn–2Nb–0.1Y alloy compressed at 0.5 s^−1^. (**a**) 1100 °C, (**b**) 1200 °C, and (**c**) 1250 °C.

Figure 4 shows the compressed microstructure of the Ti–43Al–3Mn–2Nb–0.1Y alloy obtained at different temperatures with a strain rate of 0.5 s^−1^. Figure 4a shows the microstructure of the TiAl alloy deformed at 1100 °C/0.5 s^−1^, which corresponds to the instability region. Many cracks can be observed clearly due to the low deformation temperature and high strain rate. Almost no DRX occurred. Bands of flow localization were also observed in the microstructure, which can be ascribed to the local temperature increase at a high strain rate. The heat produced during compression does not have sufficient time to dissipate, resulting in flow localization. When the temperature is increased to 1200 °C (0.5 s^−1^), some lamellae had already begun to transform into fine grains (Figure 4b). However, a few cracks can still be found in the microstructure, indicating that the deformability of the TiAl alloy is still poor, which agrees with the processing map. When the alloy was compressed at 1250 °C (0.5 s^−1^), a large number of equiaxed grains formed, while some residual lamellae still existed due to the high strain rate (Figure 4c). No cracks were observed, suggesting that the deformability was significantly enhanced by increasing the temperature.

**Figure 5:**
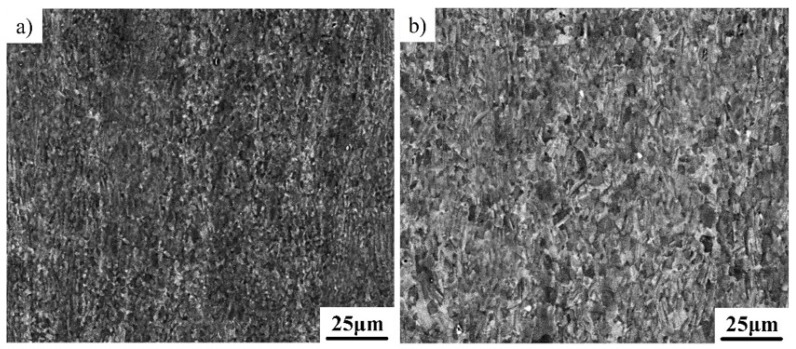
The microstructure of the Ti–43Al–3Mn–2Nb–0.1Y alloy compressed at (**a**) 1200 °C/0.01 s^−1^, and (**b**) 1250 °C/0.01 s^−1^.

In order to obtain TiAl alloys with fine grains and good ductility, it is generally anticipated that complete DRX can occur during hot deformation [36]. Thus, the microstructure of the Ti–43Al–3Mn–2Nb–0.1Y alloy compressed at the stability domain was also studied. When the alloy was compressed at 1200 °C/0.01 s^−1^, which corresponds to the peak efficiency domain in the processing map, the initial coarse lamellae had almost transformed into fine equiaxial grains as can be seen from the SEM image in Figure 5a. This indicates that the optimum deformation condition proposed by the processing map is correct. As shown in Figure 5b, fine equiaxial grains are also the main feature of the compressed microstructure obtained at 1250 °C/0.01 s^−1^. It is well known that increasing the temperature can provide a larger driving force for DRX. However, heat dissipation will increase with an increase in temperature. This is the main reason why the efficiency of power dissipation at 1200 °C/0.01 s^−1^ is higher than that at 1250 °C/0.01 s^−1^. Thus, the optimum deformation condition can be determined to be 1200 °C/0.01 s^−1^.

**Figure 6:**
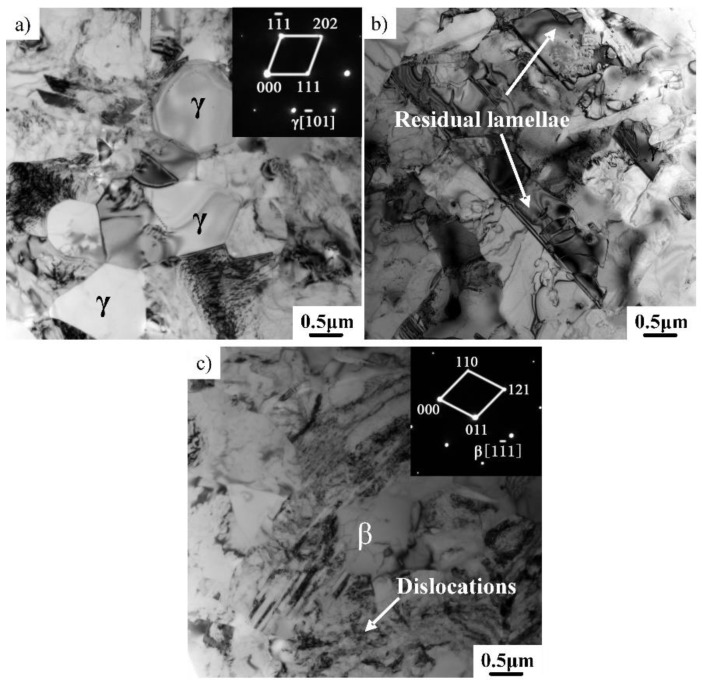
TEM bright field (BF) images of the microstructure corresponding to Figure 5a. (**a**) Equiaxed γ grains, (**b**) residual lamellae, (**c**) β phase.

To further identify the deformed microstructure and the related mechanism, the microstructure of the Ti–43Al–3Mn–2Nb–0.1Y alloy compressed at 1200 °C/0.01 s^−1^ was investigated by TEM. As shown in Figure 6a, a great deal of recrystallized γ grains can be identified using selected area electron diffraction (SAED) patterns. This confirmed that DRX is the main deformation mechanism during isothermal compression, which should be related to the stacking fault energy (SFE) of the alloy. The γ-TiAl alloy generally has a low SFE (60–90 mJ/m^2^) [37]. The SFE can be further decreased to below 52 mJ/m^2^ by the addition of Mn [38]. A low SFE can provide better conditions and foundations for DRX. A low SFE leads to larger dissociation distance of the dislocation cores, which are thus less mobile. Dissociated screw dislocations have a lower cross-slip ability as the line defects have to first reconstruct to a more compact form. As a result, low SFE results in a larger amount of dislocation pile-ups and debris, which facilitate the recrystallization process [39,40]. Although most of the lamellae had decomposed to recrystallized γ grains, some remnant lamellae can also be found using TEM (Figure 6b). The residual lamellae should be related to the orientation θ between the loading direction and the γ/α_2_ lamellae [41]. When θ = 45°, the lamellae in the soft orientation can extensively deform, which promotes the complete decomposition of the lamellae. When θ = 0° or 90°, the deformation of the lamellae in a hard orientation was limited, leading to the residual lamellae. Moreover, a small quantity of β phase can be identified, as shown in Figure 6c, which is critical for the improvement of hot workability [1]. 

### 3.4. Nearly Isothermal Forging

**Figure 7:**
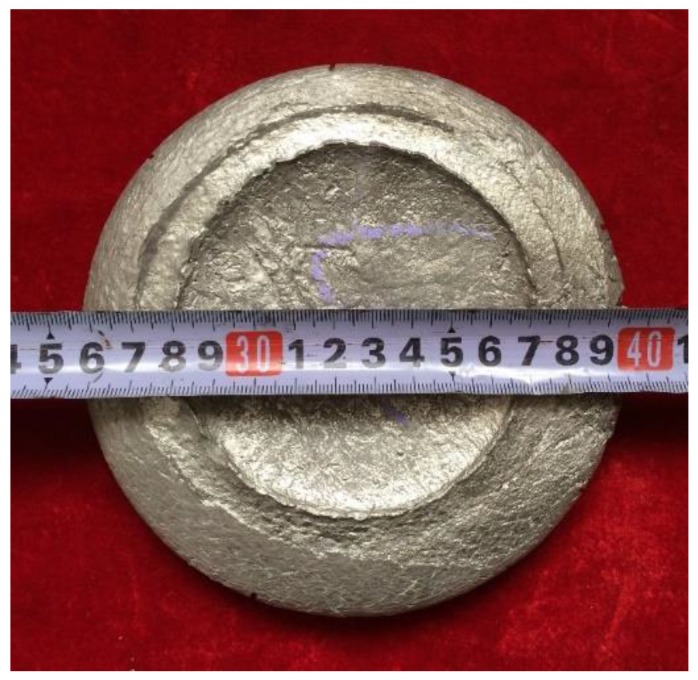
The appearance of the Ti–43Al–3Mn–2Nb–0.1Y billet.

In order to verify the accuracy of optimized processing parameters, an actual forging experiment was conducted using a four-column hydraulic machine. As shown in Figure 7, an intact Ti–43Al–3Mn–2Nb–0.1Y billet was successfully produced by one-step forging using a four-column hydraulic machine with an engineering strain of 70–80%. As known, the hot deformation of TiAl alloys is generally realized by multi-step canned forging [42,43,44]. Severe cracking tends to occur when these alloys were forged by a one-step large deformation. By contrast, the crack-free appearance of the pancake (Figure 7) further confirmed that the β–solidifying Ti–43Al–3Mn–2Nb–0.1Y alloy exhibits excellent hot workability.

**Figure 8:**
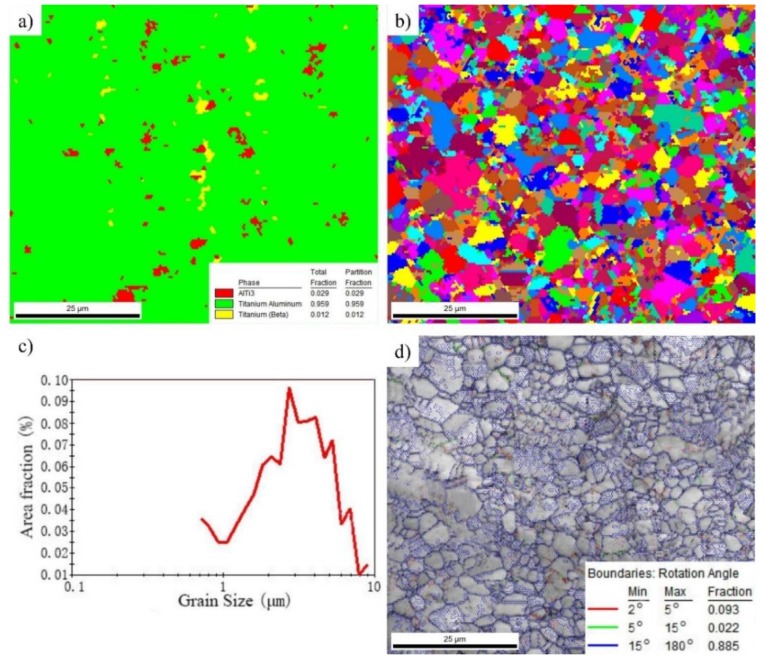
Electron back-scattered diffraction (EBSD) maps of the Ti–43Al–3Mn–2Nb–0.1Y billet. (**a**) Phase composition, (**b**) grain distribution, (**c**) grain size, and (**d**) grain boundary feature.

An analysis of the microstructure of the billet was conducted in detail using EBSD technology, as shown in Figure 8. The phase distribution map shows that the as-forged microstructure consists of 95.9% γ phase, 2.9% β phase and 1.2% α_2_ phase (Figure 8a), indicating that the initial γ/α_2_ lamellae had almost completely transformed into the γ phase. The grain distribution map (Figure 8b) shows that almost all of the grains have an equiaxed shape. Almost no residual lamellae can be identified. By contrast, for the TNM alloy, even though the alloy was deformed to 75%, a large amount of residual lamellae would still exist [45]. The grain size was measured and analyzed statistically. The statistical result (Figure 8c) shows the grain size of the alloy is generally below 10 μm, and the average grain size is about 4.9 μm, indicating that the initial as-cast microstructure had been significantly refined after a one-step large deformation. The grain boundary feature (Figure 8c) was also characterized using EBSD technology. It can be seen that the volume fraction of the low-angle boundary is very low. By contrast, the volume fraction of the large-angle boundary reached 88.5%, which further indicated that DRX plays a leading role during the forging process.

**Figure 9:**
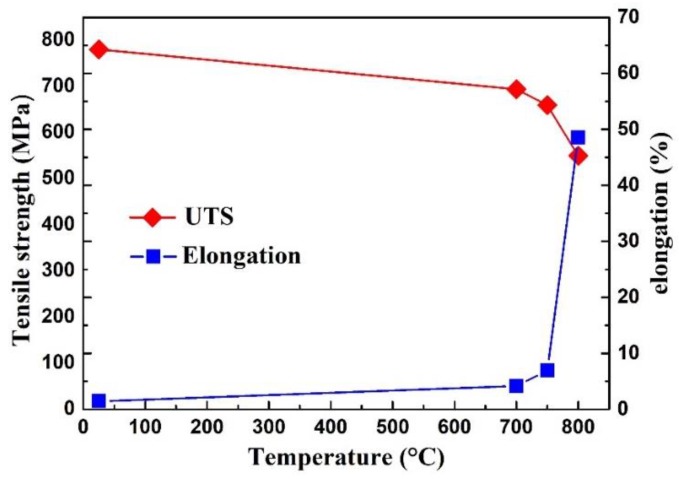
The dependence of tensile strength and elongation on temperature of the as-forged Ti–43Al–3Mn–2Nb–0.1Y billet.

Tensile tests were also conducted to analyze the effect of one-step large deformation on the ultimate tensile strength (UTS) and elongation (δ) of the Ti–43Al–3Mn–2Nb–0.1Y alloy. The as-cast ingot exhibits poor RT tensile properties. The UTS is below 500 MPa, and δ is lower than 0.8%, which can be ascribed to the coarse as-cast lamellar microstructure. The dependence of UTS and δ on temperature are shown in Figure 9. It can be seen that the UTS is 780 MPa and the δ is 1.44% at RT. The RT properties are higher than that of Cr- and Mn-containing TiAl alloys. The improvement of tensile properties was mainly due to microstructure refinement. When the testing temperature is 700 °C, the UTS and δ are 694 MPa and 4.2%, respectively, indicating that the alloy still exhibits high strength and low elongation at 700 °C. At 750 °C, the UTS and δ are 659 MPa and 6.9%, respectively. While at 800 °C, δ dramatically increased to 48%, and the UTS decreased to 549 MPa. This indicates that the ductile–brittle transition temperature (DBTT) of the current alloy should be 750–800 °C. According to previous studies, the DBTT of TiAl alloys without Nb is just 700–750 °C [20]. By contrast, the DBTT of high-Nb containing TiAl alloys can reach to more than 800 °C due to the high Nb content [46]. Thus, the DBTT of the current alloy is higher than TiAl alloys without Nb but lower than that of high-Nb-containing TiAl alloys. Above all, the Ti–43Al–3Mn–2Nb–0.1Y alloy exhibits excellent hot workability, fine as-forged microstructure, and good tensile properties.

## 4. Conclusions

In this study, the hot workability, microstructural evolution and tensile properties of a Mn- and Nb-containing TiAl alloy were systemically evaluated.

(1) The Ti–43Al–3Mn–2Nb–0.1Y alloy exhibits low deformation resistance and low deformation activation energy (392 KJ/mol). A processing map was developed. An instability region Ⅰ, occurring at low temperature (1100–1200 °C) and high strain rate (0.5 s^−1^), was identified, which is associated with cracking and flow localization. The optimum deformation condition was determined to be 1200 °C/0.01 s^−1^. This indicates that the present alloy has good hot deformability and wider hot working windows. 

(2) A Ti–43Al–3Mn–2Nb–0.1Y billet was prepared by one-step forging with a large deformation amount (70–80%). The microstructure of the billet is almost completely composed of recrystallized γ grains with large angle boundaries. The grain size of the alloy is below 10 μm.

(3) The Ti–43Al–3Mn–2Nb–0.1Y alloy has high tensile strength (780 MPa) and high elongation (1.44%) simultaneously, which benefits from fine γ grains. The ductile–brittle transition temperature is between 750 and 800 °C.
